# Application of Through Glass Via (TGV) Technology for Sensors Manufacturing and Packaging

**DOI:** 10.3390/s24010171

**Published:** 2023-12-28

**Authors:** Chen Yu, Shaocheng Wu, Yi Zhong, Rongbin Xu, Tian Yu, Jin Zhao, Daquan Yu

**Affiliations:** 1 School of Electronic Science and Engineering, Xiamen University, Xiamen 361005, China; 36120211150419@stu.xmu.edu.cn (C.Y.); 36120211150391@stu.xmu.edu.cn (S.W.); zhongyi@xmu.edu.cn (Y.Z.); xurongbin@xmu.edu.cn (R.X.); 36120200155810@stu.xmu.edu.cn (T.Y.); 2 Institute of Electronics Packaging Technology and Reliability, Beijing University of Technology, Beijing 100021, China; zhaojin@emails.bjut.edu.cn

**Keywords:** Through Glass Via, MEMS sensors, sensor manufacturing, sensor packaging

## Abstract

Glass has emerged as a highly versatile substrate for various sensor and MEMS packaging applications, including electromechanical, thermal, optical, biomedical, and RF devices, due to its exceptional properties such as high geometrical tolerances, outstanding heat and chemical resistance, excellent high-frequency electrical properties, and the ability to be hermetically sealed. In these applications, Through Glass Via (TGV) technology plays a vital role in manufacturing and packaging by creating electrical interconnections through glass substrates. This paper provides a comprehensive summary of the research progress in TGV fabrication along with its integrations, including through via formation and metallization. This paper also reviews the significant qualification and reliability achievements obtained by the scientific community for TGV technology. Additionally, this paper summarizes the application of TGV technology in various sensors such as MEMS sensors and discusses the potential applications and future development directions of TGV technology.

## 1. Introduction

Due to the progress of 5G, smart cars, medical gadgets, and other industries, electronic products are designed to be more portable and convenient. Much research has been invested in the fabrication, integration, and packaging of sensors, aiming to improve performance and dependability and cut down costs. The packaging of MEMS sensors can constitute up to 30% of the cost of producing a device [1], and the sealing capacity and interconnectivity significantly impact device performance, which subsequently affects the production and application of sensors. Wafer-level packaging, exhibiting manufacturing efficiency and excellent device performance, has advantages in reducing the size and saving costs compared to device-level packaging [2].

Through Glass Via (TGV) is the vertical electrical interconnection through the glass substrate, which corresponds to TSV. Glass substrates possess superior electrical properties and lower parasitic capacitance than common silicon and SOI substrates, facilitating the extension of high-frequency signal transmission [3]. The excellent optical properties of glass make it more suitable for optical applications such as Micro-Opto-Electro-Mechanical Systems (MOEMS) [4]. Adjusting the composition of the glass and optimizing surface treatments can modify the coefficient of thermal expansion (CTE) and mechanical strength of the substrate. This results in improved metal adhesion, stress control, and reliability [5,6]. TGV technology supports a wide range of thicknesses (from 50 µm to 900 µm) and large wafer sizes (from 6″ to 12″) and panels (from 510 × 515 mm to 1500 × 800 mm). The glass-based process is more straightforward, without the need to deposit an insulating layer on the inner wall of the TGV, making the manufacturing cost of glass package substrates much lower than that of silicon substrates. Glass can be bonded to substrates, including silicon and other glasses, using techniques such as anodic bonding and direct bonding. These methods create a stable vacuum environment suitable for inertial sensors like accelerometers and gyroscopes [1,2,5,7,8,9,10,11]. The TGV process provides the fabrication and metallization of high-density, high-aspect-ratio glass vias, enabling a reduction in device size while maintaining high-performance electrical interconnects. Intel says glass substrate technology can increase the chip area in a single package by 50%, allowing more chiplets to be crammed in. It reduces the thickness by about half compared to ABF plastic, providing higher signaling speeds and power efficiencies [12].

There are many reports and studies on TGV. However, fewer introductions and summaries are provided on applying TGV technology in the field of sensors. The critical processes of TGV technology are glass via formation and via metallization. Among them, there is a more significant amount of the review literature on glass via processing, while there are limited reviews on via metallization. This paper presents an overview of the research progress on TGV fabrication, which encompasses glass via formation and metallization, considering the advances and findings accomplished by the scientific community. The applications of TGV technology in sensors are reviewed, and the current challenges and prospects of TGV are examined.

## 2. Through Glass Vias’ Formation and Metallization Techniques

Glasses, including quartz, borosilicate, soda–lime, and high-alumina glasses, primarily consist of silica and other oxides. There is a variety of options for glass substrates in terms of the size, thickness, and material properties. Major glass manufacturers such as Corning, Asahi, and Schott offer panel glass that is oversized (over 2 m × 2 m) and ultra-thin (less than 50 µm), as well as thin and flexible glass materials [13]. Corning can provide AF32 glass with a maximum size of 12-inch wafers or 30 cm × 30 cm square substrates, with thicknesses ranging from 30 µm to 500 µm. Schott can provide BOROFLOAT Borosilicate glass with a maximum size of 230 cm × 170 cm square substrates and thicknesses ranging from 0.7 mm to 25.4 mm. Different TGV processes can process a variety of substrates, thicknesses, and diameters. In wafer-level packaging, high-quality borosilicate or quartz glass is commonly used as a substrate, with microperforations having a diameter ranging from 10 µm to 100 µm and aspect ratios ranging from 1 to 70. For certain sensor packaging and manufacturing purposes, larger sizes (more than 300 µm) and lower aspect ratios (less than 1) may still fulfill the required criteria.

As stated earlier in this review, the primary processes involved in TGV technology are the forming and metallization of vias. This section presents the established methods for through-hole formation in glass, such as the abrasive jet micromachining, electrochemical discharge machining, laser ablation, and photosensitive glass methods. Details of each processing method are provided, including the process principle, pore-forming capability, and the type of glass that can be processed. Each process is introduced with the principle, ability, and research advancements for through-hole metallization technology based on glass through-hole forming technology.

### 2.1. Through Glass Vias’ Formation Techniques

#### 2.1.1. Abrasive Jet Machining (AJM)

Abrasive jet machining is a non-conventional process whereby the surface is treated using an abrasive jet to create a specific surface shape [14,15,16,17,18,19,20,21,22,23,24,25,26]. The AJM process typically uses abrasives such as $Al_{2}O_{3}$, garnet, SiC, or diamond particles and has the capability to handle a diverse range of substrates such as metals, glass, ceramics, polymers, and composites [19,22]. The AJM process is suitable for processing large-sized and high-thickness substrates. It can create blind grooves, blind holes, and vias structures or polish the surface of materials [23]. The localized processing and protection of non-processed areas are typically accomplished by depositing a particle-resistant metal or polymer mask on the surface of the substrate [15]. AJM is a comparatively cost-effective and accurate alternative to conventional machining techniques. The non-thermal machining method does not affect material properties and can be used in non-dust-free environments. It is widely used in semiconductor manufacturing, electronic devices, microfluidic channels, and microelectromechanical systems (MEMS) [16].

The AJM process using doped abrasives can be classified into three categories based on the type of flow medium involved: abrasive slurry jetting, abrasive water jetting, and abrasive air jetting. Abrasive air jet machining is also known as sand blasting and powder blasting [27]. There are variations in abrasive flow patterns between air and slurry/water-based systems, but their erosion patterns within the glass are similar, primarily relying on brittle erosion to produce cracks and debris to remove material [20]. Processes that employ slurry as the flow medium boast a relatively small erosive footprint owing to the concentrated spray area and the low impact of the collisions between the abrasives. However, the jet quickly decelerates before reaching the substrate’s surface. At the same jet pressure, the erosion rate of ASJM is significantly lower than that of AJM [20].

AJM research in the field of glass cutting and drilling is relatively mature. $Al_{2}O_{3}$ is the primary abrasive used, with particle sizes ranging from 5 µm to 100 µm, with smaller abrasives being used to achieve lower in-hole roughness. The abrasive is propelled by compressed air, water, or slurry and comprises 1–8.2% of the mass of the jet. The impact angle, jet pressure, nozzle distance, nozzle inner diameter, and mass percentage of the abrasive in the jet constitute the main process parameters [14]. The duration of processing a single hole takes a few seconds, with the processing speed ranging between 0.1 µm/s and 32 µm/s in an air environment and between 0.6 µm/s and 4.4 µm/s in a slurry environment. It is possible to improve efficiency by processing a mask and using multiple nozzles in parallel [27]. Vias with diameters ranging from 0.3 mm to 6 mm can be produced with maximum aspect ratios of approximately 5. The jet injects from one side, resulting in tapered holes with a greater diameter on the inlet side and a lesser diameter on the outlet side. The accurate alignment and symmetrical machining of both front and back sides permit double conical vias. The shape of the bottom of the blind aperture is influenced by the air flow pressure and particle velocity, resulting in concave (U-shaped), flat, or convex (W-shaped) structures [15,25]. Direct drilling without masks produces vias nearly four times wider than the diameters of the jet, and the mask influences the bottom shape of the hole and reduces the frosted areas around the vias [21]. Ultrasonic vibration-assisted abrasive waterjets (UV-ASJ) enhance drilling performance by inducing a brittle–ductile transition mechanism that enables the material to detach from the target in a desirable manner. Additionally, UV-AJM, along with small abrasive sizes and low kinetic energy, facilitates uniform drilling that minimizes W-forms, diminishes surface roughness, and improves drilling efficiency [26]. Overall, the AJM technique is better-suited for processing low-aspect-ratio vias in large-size thick glass substrates. The research on the TGVs’ formation principle and model is relatively advanced. However, it is difficult to quickly drill, and the roughness of the vias’ inner wall is high, which makes TGV metallization hard to achieve.

#### 2.1.2. Electrochemical Discharge Machining (ECDM)

The electrical discharge method uses breakdown and high pressure to generate heat in glass. Molten glass splashes away from the substrate, forming vias in glass [28]. Vias’ inner wall is fire-polished until very smooth, and residual stresses are removed by post-processing annealing. This method can drill vias in fused quartz, soda–lime, and non-alkali glass without requiring a mask or dust-free environment. The electrical discharge method is more efficient than machining, completing each via in just 0.2 s–0.5 s, and can be simultaneously executed through numerous discharge ports. It is possible to drill vias in glass with thickness from 100 µm to 500 µm. Thick glass produces more molten glass splash during drilling; thus, thin glass with thickness from 100 µm to 200 µm is preferred. The electrical discharging method allows for a high degree of process freedom, enabling the processing of small, fine-pitch via arrays. Vias with a diameter of 20 µm and a spacing of 60 µm can be fabricated on thin glass, while vias with a top diameter of 60 µm and bottom diameter of 40 µm can be produced on thick glass.

Electrochemical discharge machining, or spark-assisted chemical engraving (SACE), is a machining technology that hybridizes electrochemical machining and electrical discharge machining [29,30,31,32,33,34,35,36,37,38,39]. The ECDM method can achieve the parallel processing of large-area vias arrays by customizing the working electrode, inheriting the high-cost effect and process flexibility of the EDM method. The diameter and morphology of vias processed by ECDM are related to the size and surface roughness of the tool electrode. When the electrode size and surface roughness decrease, the heat-affected zone and overcut decrease [32]. The average material removal rate was 3 µm per second for drilling and 50 µm per second for machining. The electrode widths used ranged from 80 µm to 150 µm, and drilling typically involved an overcut from 150 µm to 300 µm on the front side and from 100 µm to 200 µm on the back side, resulting in a lower limit of 280 µm–300 µm for vias [37]. By adding magnetic stirring to the discharge process, the bubble aggregation and erosion effects can be optimized, resulting in an increase in the aspect ratios of vias and a reduction in the heat-affected region [39]. The electrochemical discharge method is a low-cost, small-scale process. However, the drilling rate is slow, and the process is random. It is difficult to achieve a vertical via, and the presence of heat-affected areas affects reliability.

#### 2.1.3. Photosensitive Glass

Alkali and alkaline-earth metal oxides are added as cosolvents to $SiO_{2}$ glass, along with metals like Al and photosensitive active ingredients like Au and Ag, to produce photosensitive glass. Reactions occur within photosensitive glass after exposure to light of specific wavelengths. Subsequently, the glass undergoes permanent property modifications after being exposed to heat treatment. Chemical etching enables the production of glass microstructures [40]. Commonly used photosensitive glass, such as Schott’s FOTURAN II photosensitive glass, undergoes a photochemical reaction to release free electrons when exposed to UV light. Heating the glass to 500–600 °C causes doped Ag+ within the glass to absorb electrons and convert into silver atoms, becoming a nucleation center to develop lithium metasilicate crystals ($Li_{2}SiO_{3}$). The hydrofluoric acid etching rate of $Li_{2}SiO_{3}$ reaches 20–50 times higher than that of the unaffected zone, enabling the selective etching of the glass and unveiling microstructures like vias [41].

**Figure 1:**
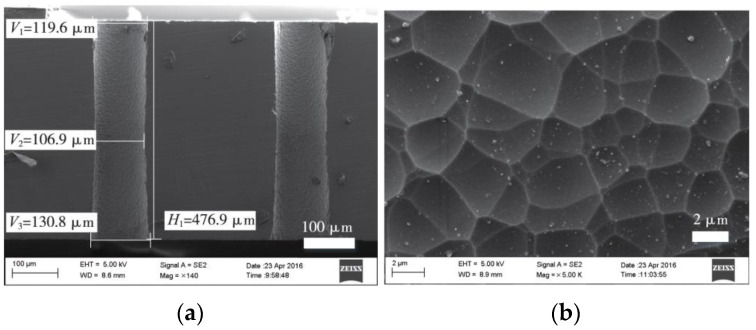
SEM image of photosensitive glass via after wet etching: (**a**) cross-sectional view; (**b**) surface view of inner wall [45].

Photosensitive glass can be modified by UV laser irradiation or maskless modification. Both processing methods allow the formation of vias with aspect ratios of more than 8 (from 25 to 35) [42,43,44], with good perpendicularity (inclination as low as 1°) and roughness (less than 1 µm) of the inner wall of the vias [45], as illustrated in Figure 1 [45]. Brokmann et al. [46] processed glass by plasma etching and compared it to wet etching methods, demonstrating that plasma etching offers new degrees of freedom in microstructure control and microsystem fabrication. Photosensitive glass shows promise for high-density interconnection and microsystem integration but faces challenges such as the high cost of photolithography and laser-induced processes, the high cost of glass, and the complexity of processing.

#### 2.1.4. Glass Reflow Process

**Figure 2:**
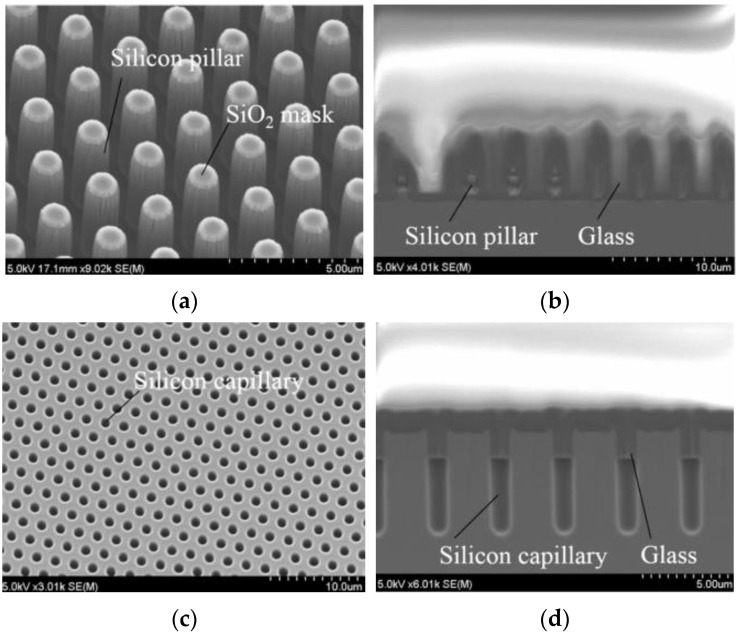
Glass reflow process into small cavities. (**a**) Silicon pillar mold; (**b**) silicon capillary mold; (**c**) penetration depth of pillar mold under third reflow condition; (**d**) penetration depth of capillary mold under third reflow condition [48].

The glass reflow process capitalizes on the liquidity of molten glass, enabling its flow into reserved spaces to forge the intended structure [47]. The process typically employs Deep Reactive Ion Etching (DRIE) to treat the silicon substrate and create a reverse structure. The silicon substrate is then anodically bonded to the glass substrate, resulting in a confined cavity. Afterward, the bonded wafer is heated to melting temperature in an annealing furnace, and the molten glass is drawn into the silicon cavity until the space is completely filled with glass, as shown in Figure 2 [48]. The silicon–glass substrate is created by thinning and polishing the reflowed substrate to remove a specific amount of glass and silicon on either side. Glass microstructures, such as rings, gears, tuning forks, etc., may be produced using the glass reflow technique [49]. In the realm of wafer-level packing, low-resistance silicon can be depended on for vertical electrical connections, while glass provides signal insulation and device protection [50].

The glass reflow technique was applied in the fabrication of glass micro-lens arrays [51] and hermetically sealed integrated silicon through-mechanical sensors [52]. The study by Haque et al. [47] employed the glass reflow process to produce package substrates suitable for hermetic sealing and galvanic isolation, demonstrating the viability of the process for use in capacitive pressure sensors and hermetically sealed resonators. Toan et al. [48] addressed the difficulty of filling tiny scale patterns with glass and investigated the phenomenon of glass reflux into large cavities, microgrooves, and microcapillaries. They demonstrated that the addition of a 50 nm $SiO_{2}$ film on the surface of a silicon substrate can enhance the surface’s wetting properties with glass. Due to the confined space and high surface tension present in microcapillaries, filling the glass is challenging. Extending the reflux time helps enhance the filling capacity of reflux [53]. Li et al. [54] employed a double-sided reflow procedure to address the cavity formation issue that arises after glass reflow. Meanwhile, Liu et al. [55] utilized nano-glass powder as a substitute for glass substrate to enhance the filling effect and prevent the anodic bonding and thinning process. Nguyen et al. [56] used glass reflow to fabricate CMUT arrays, proving that the process can be applied to optical microfluidics, 3D-MEMS, etc. Kuang et al. [2,10,11] created TGVs in a silicon substrate using the glass reflow method. The triple anodic bonding of the TGV substrate, the MEMS structure, and the glass cap completed the wafer-level vacuum packaging.

#### 2.1.5. Laser Ablation (LD)

Mechanical techniques including the abrasive jet machining method make it difficult to form vias less than 100 µm in diameter. In contrast, laser ablation presents an effective means of creating low diameter and high depth-to-width ratio vias by forming micro-vias through thermal shock and ablation [57,58,59,60,61,62,63,64,65,66,67,68]. Lasers have widespread applications in cutting and drilling various materials, including PCB substrates. The research on the laser processing of glass substrates is highly developed. The commonly employed lasers for glass substrate processing include infrared CO_2_, ultraviolet UV-YAG, and ArF excimer lasers. Laser ablation is a production process that requires no mask and enables quick processing, making it suitable for mass production. Yet challenges such as heat-affected area expansion, hole thermal damage, and the emergence of protrusions near vias’ edges impede bonding [59]. Optimization can be achieved by various means, including the addition of an organic layer on the glass surface to reduce laser damage, immersion of the glass in a cooling liquid to decrease the thermal impact, preheating the glass before processing, or the use of shorter pulse lasers.

The absorption rate of glass in the visible range is low, so UV and IR lasers prove to be more efficient for glass processing [67]. However, even within the visible range, ultrashort pulsed lasers such as picosecond and femtosecond lasers can be used to create vias by enhancing the absorption of glass through multiphoton absorption. The non-thermal processing of short pulsed lasers mitigates thermal damage within the glass, though the development of stress waves can create microcracks in the internal wall of the hole [62]. CO_2_ lasers have long been one of the most commonly used lasers in industry due to their relatively low cost and simplicity of equipment. CO_2_ lasers are capable of producing vias with diameters of less than 100 µm and spacing of 400 µm on 500 µm Schott D263Teco glass [58]. The CO_2_ laser is capable of achieving through-hole fabrication on a 140 µm thick polymer-laminated glass with an incident surface diameter of 120 µm and an output surface diameter of 75 µm [64].

The processing speed of picosecond lasers is 500 times faster than that of CO_2_ lasers, reaching 10 m/s. After a latency period of about 1 µs, the glass reaches the threshold temperature by multiphoton absorption, at which the irradiated material is rapidly heated by linear absorption to form vias with a diameter of 10–20 µm [67]. Using liquid-assisted laser processing, CO_2_ lasers are capable of producing arrays of vias ranging from 100 to 200 µm without any crack or burn zones when operated at 6 W power with a scanning speed of 11.4 mm/s [57]. In order to avoid defects in the 500 µm damage zone, picosecond lasers utilize liquid-assisted processing. It was demonstrated that machining 100 µm diameter vias in 800 µm thick glass can be achieved with a reduced heat-affected zone of 15 µm and a reduced taper of 2 µm [66]. The addition of a PDMS protective layer to the glass surface reduces the thermal effect on the glass surface bumps from 15.1 µm to a minimum of 1.2 µm [59].

#### 2.1.6. Laser-Induced Deep Etching (LIDE)

Several previously discussed drilling methods have problems such as the inability to process small holes, poor accuracy, and cracking. To address such shortcomings, LPKF put forth a high-precision, low-cost laser-induced deep-etching (LIDE) technique [69]. Use a picosecond laser to process a glass substrate to form induced areas with diameters of approximately 1 µm [70]. Place the glass in hydrofluoric acid or alkaline solution; the etching rate of the laser-induced areas is much higher than other areas of the glass, and the laser-induced region is enlarged to form vias and remove thermal damage. LIDE is suitable for processing vias or arrays of blind holes of arbitrary size and spacing. It is capable of creating cavities of any shape or large vias by closed connecting closely spaced vias (1–10 µm) [71].

**Figure 3:**
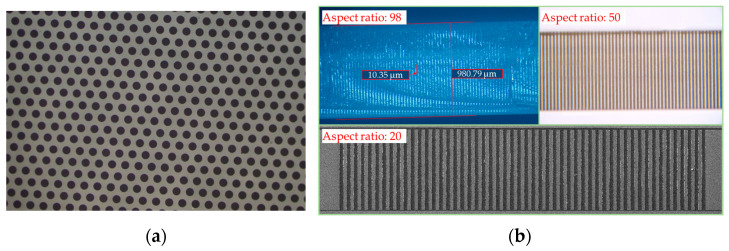

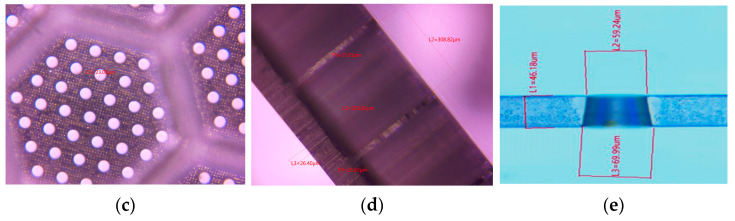
TGV samples processed by Xiamen Sky-semi: (**a**) the top view of glass through-hole array; (**b**) the cross-sectional view of ultra-high-aspect-ratio (from 20 to 100) glass vias; (**c**) the top view of slot–vias structure; (**d**) the cross-sectional view of slot–vias structure; (**e**) the cross-sectional view of trapezoidal via [78].

The LIDE method is recognized as a highly potential technology for TGV production [72]. LIDE is well-researched [73,74,75,76] and has been extensively adopted by industrial players, including Corning, Schott, AGC, Mosaic Microsystems, LPKF, Plan Optik, Samtec, and Xiamen Sky-semi. Typical glass vias exhibit an hourglass shape with diameters ranging from 20 µm to 100 µm and substrate thicknesses ranging from 50 µm to 1 mm. The aspect ratios typically range from 5 to 10. Glass vias feature smooth sidewalls (Ra < 0.8 µm) without cracks and taper angles ranging from 0.1 to 30°. The glass surface is smooth after etching, with roughness less than 20 nm. The etching rate of the alkaline is lower compared to that of the acid; however, the induced region’s selective etching nature is better, allowing the formation of near-vertical vias with high aspect ratios. Quartz glass and borosilicate glass show higher selective etching ratios than other glass types [77]. It is possible to process glass vias with diameters less than 7 µm and aspect ratios as high as 50, 70, and even 100. The integration of vias and blind holes with different diameters on the same glass substrate can be realized by multiple LIDE processes or cavity–vias structures, as shown in Figure 3. The LIDE process takes longer to process large-size cavities. It requires machining blind holes with set point spacing, which are connected to form cavities. The processing time of cavities depends on the laser-induced dot spacing and the laser travel speed. The larger the dot spacing is, the higher the roughness at the bottom of cavities (Ra > 0.1 µm) is, while too small dot spacing can cause heat build-up problems inside the glass during laser induction, affecting the processing results. The LIDE method has multiple advantages in speed, quality, and cost. It can process wide ranges of TGVs with high compatibility with other processes, which has high potential for application in the fields of 3D integration and wafer-level packaging.

### 2.2. Through Glass Vias’ Metallization Techniques

#### 2.2.1. Conductive Paste Filling Method

Metallic conductive pastes offer adjustable CTE and can be directly bonded to glass, which is compatible with the TGV process. Nano-silver paste and conductive copper paste can be chosen as materials for filling vias. Conductive paste can be filled into TGVs through screen printing and sintering to construct electrical interconnects [79] or using the 3D printing process: using small-sized nozzles to spray out conductive paste, while using the laser to sinter the paste to form an electrical connection. A vacuum environment or evacuation can avoid the voids inside the paste [80,81]. The conductive paste filling method can achieve TGV array metallization with high aspect ratios and densities. There may be a volume reduction in the paste after curing results in dishing (from 5 µm to 20 µm, depending on the via diameters and substrate thickness) in the vias, which can be removed by refilling the paste or grinding the glass substrate [82]. There remains a residue on the surface of the glass after the printing and filling process. After sintering, it is necessary to polish the front and back of the glass to enhance its surface cleanliness.

**Figure 4:**
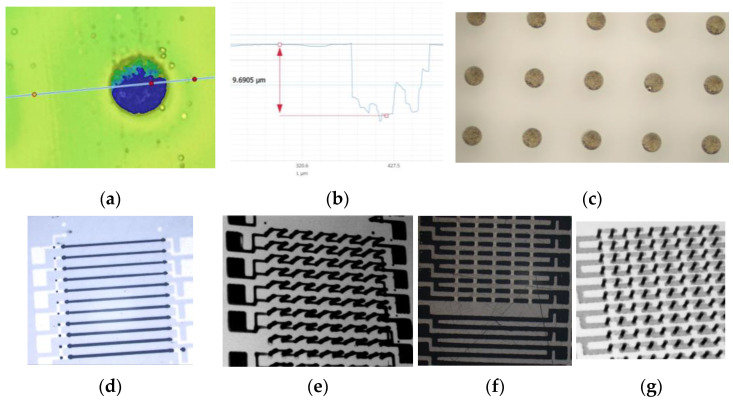
(**a**) The 3D images of paste-filled vias; (**b**) dishing of the paste-filled vias before thinning; (**c**) the top view of paste-filled vias after thinning; (**d**) the OM image of daisy chains using 3D printing to fill vias and surface wiring; (**e**) the X-ray image of daisy chains using 3D printing to fill vias and surface wiring; (**f**) the OM image of daisy chains using vacuum plugging to fill vias and surface wiring (thinning causes cracks in the glass); (**g**) the X-ray image of daisy chains using vacuum plugging to fill vias and surface wiring.

There is more research on the application of the conductive paste filling method in TSV metallization but less research on the application of TGV metallization. Takahashi et al. [28] achieved the filling of conductive copper paste through 3D printing and screen printing inside TGVs with a 50 µm diameter and a 130 µm pitch, where the resistivity of the copper paste was about 1.6–1.9 Ω·m/sq. Our team successfully filled 50 µm and 100 µm diameter vias on a 400 µm thick BF33 glass substrate using conductive silver paste. Then, we processed the daisy chain and coplanar waveguide to analyze the interconnect performance. The filled vias were free of defects such as air bubbles, and the resistivity of the silver paste inside the vias was $2.56\times 10^{-7}$ Ω·m. The conductive paste has the potential to attain an aspect ratio exceeding 10 for sealing vias and interconnections, as well as filling vias ranging from 50 µm to 1 mm in diameter, thereby making it an economical and high-performance metallization technique, as illustrated in Figure 4. However, the glass surface needs to be grinded after the paste is sintered, leading to reliability issues with the glass. The conductivity of the paste is low, and its resistivity varies during the sintering process. Therefore, the conductive adhesive filling method is suitable for applying TGV metallization with low electrical interconnect standards or very high aspect ratios, but the process stability still needs to be improved.

#### 2.2.2. Magnetic Self-Assembly Method

The widely utilized super-conformal electroplating process presents challenges in filling vias with rough sidewalls and poor hole wall morphology. The optimization of via filling for diverse aspect ratios and tilt angles is challenging. Therefore, Laakso et al. [83] proposed a solution to fix the nickel wire inside the glass vias using a magnetic-field-assisted self-assembly to achieve electrical interconnection.

Cut nickel wires can be magnetically loaded into the glass vias. The vias were filled with spin-on-glass and cured with methyl siloxane. The voids in the vias were refilled due to shrinkage, and the magnets were removed from the bottom of the substrate. The SOG was cured, and after the front-side wiring had been completed, a backside wet etch was used to thin the wafer and remove the nickel wire, thus finalizing the glass vias’ metallization process. This method enables the metallization of TGVs with high density, a high aspect ratio, and a low resistance value, while ensuring compatibility with various inner wall morphologies and roughness vias. Adjusting the composition of metal rods and SOGs can mitigate the thermal mismatch problem in TGVs. The process has strict requirements for magnetic control, which may require multiple magnets to completely fix the wires inside the vias, and the length of the magnetically controlled wires must be greater than the width to achieve proper orientation. However, using the HF etching approach to thin the wafer for exposing the nickel wires poses a challenge, with the etchant penetrating the sidewalls of the SOG and TGV vias. To overcome this, a mechanical mask can be implemented with CMP, which does not affect the thinning capability.

#### 2.2.3. Electroplating Method

**Figure 5:**
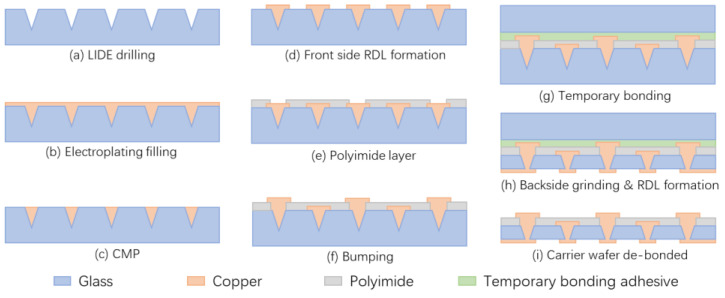
Schematic fabrication process for glass interposer.

Like the use of electroplating technology to fill TSV structures, metal can also be deposited into TGV structures through electroplating. As illustrated in Figure 5, the specific process is as follows: (1) deposit a diffusion barrier layer and seed layer in TGV pores by physical vapor deposition (PVD) and other methods; (2) fill the required metal from bottom to top by electrochemical reactions, usually by electroplating Cu; (3) remove excess Cu from the surface by methods such as wet corrosion and CMP. The bottom-up plating technique initiates from the hole’s bottom, preventing void formation during filling. However, the process is time-consuming and costly. Subsequently, partial filling methods have been introduced, which are also recognized as conformal filling. The TGV hole does not necessarily require complete filling. Instead, it can be filled along the sidewall or a semi-enclosed structure, such as a copper bridge [84], to improve the electrical connection. A resin film can be laminated as an insulation layer in the TGVs [85]. Partial filling technology can be compared to full filling technology in terms of electrical performance [86], and the electroplating time and costs are optimized to some extent.

**Figure 6:**
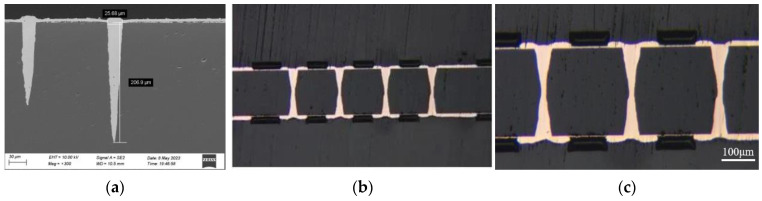
Cross-sectional SEM photos of (**a**) electroplated filled blind glass vias and (**b**,**c**) electroplated filled TGVs.

ALD demonstrates superior microporous filling performance compared to PVD and can be effectively utilized in the TSV process to deposit the insulation, barrier, and seed layers. Blind silicon vias with a 3 µm diameter and a depth of 50 µm (aspect ratio 15) can be filled using chemical plating and electroplating [87,88,89,90,91,92]. We deposited Ru as a seed layer and achieved the defect-free filling of small silicon blind holes as small as 3 µm in diameter and 45 µm in depth. Our team achieved blind glass vias’ electroplating with an aspect ratio of 7 and double-sided TGVs electroplating with an aspect ratio of 4 in collaboration with Xiamen Sky Semiconductor Technology Co., Ltd in Xiamen. Cross-sectional SEM photos of electroplated filled blind glass vias and TGVs are illustrated in Figure 6. Lee et al. [93] achieved a bottom-up electroplating process without seed layers to achieve the pore-free filling of TGV holes with a high aspect ratio, smooth sidewalls, and vertically interconnected RF MEMS devices. In order to achieve a seedless electroplating process, they used low-resistance silicon as the substrate, and the etched silicon surface can act as a seed layer. Tanaka et al. [94] plated TGV vias with a depth of 300 µm, a diameter of 60 µm at the bottom, and a diameter of 40 µm at the top to achieve partial filling and investigated the feasibility of conformal filling through the simulation analysis of the current distribution. In coplanar waveguides (CPWs) prepared on this basis, the insertion loss due to TGV is 0.2 dB at 30 GHz, and, therefore, the transmission line using TGV can be used for high-speed signal transmission using high-frequency frequency bands. Wang et al. [6] realized X-shaped partially filled TGVs and RDLs by double sides Cu conformal electroplating process. No cracks or protrusions were observed at critical locations in 100 thermal cycling experiments between −40 °C and 125 °C. This indicates that the electroplating-filled TGV process has a good application prospect for the preparation of MEMS devices.

## 3. Application of TGV for Sensors’ Manufacturing and Packaging

Packaging serves to isolate sensitive and fragile internal and external environments, protect internal space, and facilitate signal transmission. The cost of packaging MEMS sensors accounts for more than 30% of the total manufacturing cost. The optimization and enhancement of the packaging process can assist the sensors in achieving superior performance whilst reducing costs.

**Figure 7:**
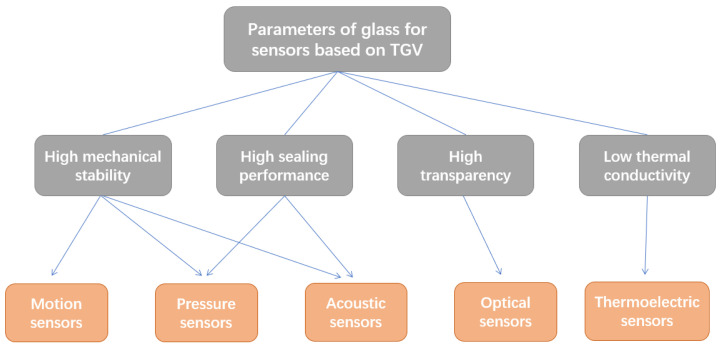
Parameters of glass for sensors based on TGV technology.

The packaging process is essential for sensors, particularly MEMS sensors. Wafer bonding technology and vertical interconnect technology are the key technologies for wafer-level packaging technology, which is of great value in achieving a smaller device size, lower manufacturing cost, and lower power consumption [10]. Glass has been widely used in sensors’ packaging due to its unique properties such as high mechanical stability, high sealing performance, high transparency, and low thermal conductivity. It can serve as a cap substrate to create a highly vacuum-sealed environment through anodic bonding, direct bonding, or metal bonding with silicon, SOI, glass, or other substrates in sensor packaging. Moreover, the development of TGV technology, mainly including vias’ formation and metallization, has made glass substrates more preferred in sensor packaging due to their superior performance advantages. As shown in Figure 7, this section provides detailed information on the application and performance of the TGV process for different types of sensors according to the performances of glass.

### 3.1. Motion Sensors

Motion sensors are typically used for motion detection and acceleration measurement. Chips require sufficient impact resistance to protect the internal microstructure. Glass has strong mechanical properties that can improve the impact resistance of sensors. TGV technology has great potential for motion sensing applications.

Ma et al. [5] used a glass adapter plate as a top cover to solve the stress problem of TSV technology. A symmetrical sandwich structure for MEMS inertial sensors based on the bulk silicon process was constructed. Blind vias were formed on a 400 µm thick glass substrate by powder blasting, and TGVs were formed by backside grinding and polishing. Al was deposited by sputtering to provide electrical interconnections in the TGVs. A redistribution layer was formed on both sides of the adapter plate by photolithography. BCB was used as an adhesive layer to bond the adapter plate to the MEMS accelerometer wafer for the wafer-level packaging of the accelerometer.

Fu et al. [7] proposed a comb structure accelerometer, in which the glass cover plate with TGV was bonded to the accelerometer anode to form a sealing and interconnection structure. Laser ablation was used to create square vias with 300 µm thickness on BF33 glass. The TGV-metallization process was performed using metal mold method [95]. Yang et al. [8] used the powder blasting method to fabricate biconical vias with a diameter of about 600 µm on a 500 µm thick glass substrate. Cr/Cu was sputtered onto the substrate as a seed layer, which was then filled with TGVs through a PPR copper plating process. Following polishing, the TGV substrate was obtained, from which shock threshold sensors were fabricated. During testing, reliable switching signals were obtained at a shock velocity of 1000 g.

**Figure 8:**
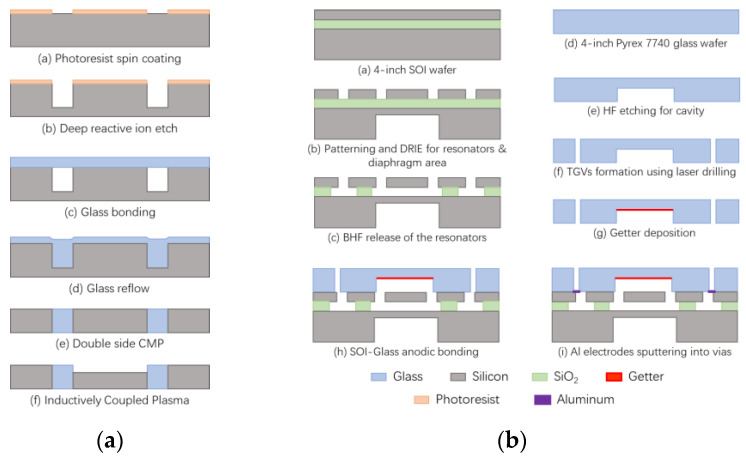
The fabrication and packaging process of (**a**) glass caps for bonding with capacitive gyroscope [9]; (**b**) the resonant pressure sensors [96].

Yang et al. [2] processed Pyrex 7740 glass using a picosecond laser at a wavelength of 532 nm and obtained vias with an entrance diameter of 90 µm, an exit diameter of 48 µm, and a depth of 300 µm. Vertical electrical interconnections were achieved using electron beam evaporated metal filler to cover the inside of the TGV, and the TGV substrate was bonded to the gyroscope to complete the package. The devices were tested to maintain a vacuum of 1 Pa for over two years. Zhang et al. [9] fabricated glass caps with low-resistance silicon as the conductive column using a glass reflow process and completed the hermetic encapsulation of the capacitive gyroscope by anodic bonding. The quality factor of the tested device exceeded 220,000, which is an order of magnitude greater than that of the unencapsulated gyroscope; the process flow of glass caps is shown in Figure 8a. Kuang et al. [2,10,11] conducted an initial investigation into the process mechanism of glass reflow, followed by the successful sealing of a gyroscope using this method. This proved the feasibility of wafer-level vacuum packaging using TGV technology in combination with triple-anode bonding.

### 3.2. Pressure Sensors

Pressure sensors can be categorized as capacitive pressure sensors, piezoelectric pressure sensors, or resonant pressure sensors, which can be used in aerospace inspection and atmospheric pressure sensing. These sensors are typically vacuum-sealed, such as micro-resonators, where the mechanical quality factor deteriorates with increasing ambient pressure due to air damping effects. The potential of the TGV process for applications such as capacitive pressure sensors has been demonstrated. Haque et al. [97] produced capacitive pressure sensors using the glass reflow method. The wafer was heated inside a tube furnace, and the grooves were filled with melted glass to form silicon conductive vias. This process completed the sealing of the sensor and provided electrical lead-in, eliminating the need to install bond wires on the front.

Kim et al. [47] used glass reflow technology to fabricate silicon–glass structured wafers for electrical interconnects, proving that glass provides good electrical isolation and minimizes parasitic capacitance. Zhenyu et al. [96] used laser drilling in Pyrex 7740 glass to fabricate glass caps. The resonant pressure sensors were fabricated by combining glass caps with TGVs on SOI wafers. The glass cap with TGV achieved both vacuum sealing and electrical lead-out, which can be seen in Figure 8b. The manufactured micro-pressure sensor was verified to have a Q-factor greater than 22,000 and was stable for 5 months, confirming the reliability of the vacuum package and electrical connection.

### 3.3. Acoustic Sensors

Changes in the external environment (such as temperature, pressure, humidity, etc.) have an impact on the properties of sensor materials, thereby affecting the propagation characteristics of sound waves (mainly the sound speed). By utilizing this principle, changes in the sound speed can be detected to determine changes in the external environment.

Chen et al. [78] designed a new three-dimensional wafer-level packaging (3-D WLP) solution to improve the performance and reliability of surface acoustic wave (SAW) filter packages with large cavities. Glass capping and vertical interconnects were realized using TGV, which can avoid the outgassing problem and prevent the contamination of the interdigital transducers (IDTs).

The application of TGV technology in acoustic sensors is still relatively limited, mainly focusing on CMUT. Ultrasonic transducers have potential applications in the medical and underwater exploration fields [98]. Capacitive micromachined ultrasonic transducers (CMUTs) offer an alternative to piezoelectric technology for producing two-dimensional ultrasonic transducer arrays with typical integrated circuit manufacturing processes. CMUT arrays can be integrated with front-end ICs through flip-chip bonding and TSV processing. TSV processing is a complex procedure with high parasitic capacitance and roughness, which can cause additional stress and degrade CMUT performance. As a result, TGV technology presents itself as a promising alternative.

**Figure 9:**
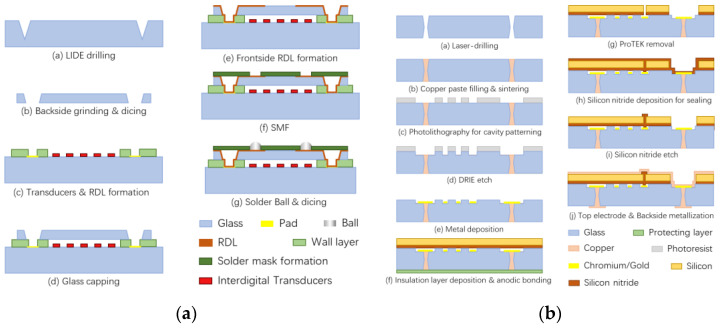
The fabrication and packaging process of acoustic sensors based on TGV technology: (**a**) SAW filter [78]; (**b**) CMUT [99].

Zhang et al. [99] developed a procedure to create vacuum-sealed CMUTs through anodic bonding on borosilicate glass substrates. They then extended this process using TGV interconnects. Laser ablation was employed to generate via holes, with 70 µm diameter at the entry and 50 µm at the exit on 700 µm thick borosilicate glass. Copper paste was used to fill the vias, which were subsequently sintered and polished to form the TGVs. CMUT array fabrication was completed, and performance tests were conducted to demonstrate the devices’ basic functionality, although they were not vacuum-sealed. Zhang et al. [3,99,100] reported a fabrication process for vacuum-sealed CMUTs on borosilicate glass substrates using anodic bonding and completed the fabrication process as shown in Figure 9. A process of fabricating using sacrificial etching was demonstrated to overcome the restriction of employing glass substrates that are compatible with anodic bonding.

### 3.4. Optical Sensors

There are still limited optical components and systems utilizing the wafer-level packaging method. Glass, which has remarkable optical and electrical characteristics, is particularly appropriate for packaging optical sensors. Brusberg et al. [101,102] used a laser drilling process to fabricate TGVs in D263T glass for 3D interconnections and integrated Mach-Zehnder interferometer (MZI) waveguides, fluidic channels, optoelectronic elements, and silicon dies to form optical sensor. Stenchly et al. [4] introduced a modular packaging system suitable for optical components and systems. The system comprised a TGV interposer and a glass cover plate. A specialized process can fabricate an optical window on the glass cover plate, achieving tilting by way of structural tilting through thermally induced stresses during high-temperature baking. TGV adaptor plates can be integrated into a glass adaptor plate body through techniques like glass reflow, with low-resistance silicon or copper serving as the interconnect material. Optical sensors or laser diodes can be packaged by bonding the glass cover plate and adapter plate. This modular design has sizable industrial potential and can be utilized in various applications.

### 3.5. Thermoelectric Sensors

Based on the adjustable thermal conductivity, glass has attracted considerable attention in the application of thermoelectric sensors. Thermoelectric sensors can convert temperature changes into electrical changes, allowing them to be applied to fabricate thermoelectric generators and wind sensors.

Thermoelectric generators can transform temperature difference or low-grade waste heat into electricity. This has a great application prospect in portable electronics, wireless sensors, and medical devices. As glass boasts low thermal conductivity, it can effectively increase the temperature difference between the hot and cold ends of micro-thermocouples, enhancing the thermoelectric conversion capability, in combination with high-aspect-ratio TGVs. Liu et al. [103] fabricated micro-thermoelectric generators based on Bi_2_Te_3_ and Sb_2_Te_3_ using 200 µm thick glass substrates. The vias were formed by laser ablation, and Bi_2_Te_3_ and Sb_2_Te_3_ were deposited in the vias at both ends of the device. The 200 µm thick glass was capable of generating a temperature difference of 138 K, which, in turn, provided an output voltage of 40.89 mV and an output power of 19.72 µW. Compared to photoresist masks, thermal sensors produced on glass substrates offer technical and cost benefits by allowing for more flexible control of the device’s output voltage and power. Wind sensors are commonly used in areas such as agricultural production, transportation, and energy harvesting. The wind force can be estimated by detecting subtle temperature changes caused by the wind with sensors. Miniaturized hot air sensors offer high initial sensitivity and low processing costs, but their reliance on heating for measurement results in high energy consumption. Glass is a low-thermal-conductivity material that can effectively reduce heat loss. Relying on TGV technology allows for electrical connections without the need for external wiring, meeting the requirements of reliability and high performance. Zhu et al. [104,105,106,107] proposed a thermal wind sensor packaging scheme, implemented using glass reflow technology. Drawing upon the performance benefits of the glass substrate and TGVs, the total heating power consumption of the sensor amounted to a mere 14.5 mW.

## 4. Discussion

Wafer-level packaging offers considerable benefits for sensor packaging, and the TGV process is capable of achieving interconnection and bonding while maintaining reliability and process compatibility [27]. TGVs also allow for greater flexibility in establishing power supply and signal transmission rules, including the seamless integration of optical interconnections, capacitors, inductors, and other devices. Intel asserted that TGV technology will redefine chip packaging boundaries, offering transformative solutions for data centers, artificial intelligence, and graphics construction, propelling Moore’s Law’s advancements.

There are several methods of drilling glass, including mechanical techniques, wet etching, and laser methods. Mechanical drilling is considered the most straightforward form of processing. This method is also economical, making it an ideal choice for processing micro-holes with low aspect ratios (near 1). By using fine drill bits and low-speed abrasives, high aspect ratios (e.g., 5) and the production of fine holes (low than 100 µm) can be achieved. The vias’ inner wall is rough (around 1.6 µm), and it takes on a conical shape when mechanically processed. The hole-forming effect can be optimized through the addition of an ultrasound or by utilizing a mix of other methods.

The electrochemical machining method, along with the focused discharge method, employs chemical etching, resulting in higher machining efficiency (50 µm/s) in comparison to mechanical machining. It is commonly used for machining vias with a thickness exceeding 300 µm and a diameter greater than 280 µm, with aspect ratios ranging from 1 to 5. The vias created through the ECDM method are conical, and the diameters are closely tied to the electrode being processed. Discharge-generated heat leads to the thermal damage to the vias and the splashing of melted glass. Optimizing the process may be possible by incorporating magnetic stirring in various ways.

Laser ablation, laser-induced deep etching, and photosensitive glass techniques have the potential to produce vias’ arrays with an ultra-high aspect ratio (over 10) and a smaller size (<100 µm), rendering them more fitting for wafer-level packaging. The production capacity of the laser ablation method is dependent on the type of laser used. There are issues related to thermal stress and stress waves in vias. The heat-affected region and debris resulting from laser ablation can be mitigated by the application of a protective surface layer or liquid processing. The processing technique of photosensitive glass is intricate, and the cost of the glass is high. However, it can achieve the processing of various microstructures and any size of holes and grooves and has high process compatibility. The LIDE process is highly suitable for high-aspect-ratio TGVs’ processing and has a wide range of commercial applications. The current development direction of LIDE is the processing of TGVs with smaller diameters, higher aspect ratios, and wider ranges of glasses.

The glass reflux technique is often used to process motion sensors or MEMS devices such as accelerometers and gyroscopes. It enables the production of packaging substrates that meet the requirements of vertical interconnection, isolation protection, and bonding. While molten glass exhibits an excellent filling ability, the capillary structure poses a challenge due to issues arising from the surface tension. The process flow for the glass reflux method typically comprises anodic bonding, annealing, and double-sided thinning. The substrate requires a high level of surface cleanliness, and the processing is relatively intricate. Optimization methods, such as replacing glass substrates with glass powder, can reduce the process flow and save costs. The comparison of TGV-formation processes is shown in Table 1.

The TGV-metallization process resembles the TSV-metallization process but is simplified, as it does not require a deposition barrier layer. The paste-filling technique is appropriate for filling ultra-high verticality vias (with an aspect ratio of over 10) with low-electrical-performance demands. The paste-filling method displays inferior stability, sealing, and process compatibility compared to the electroplating method. However, it is a cost-effective solution and provides excellent adhesion to glass. In contrast, electroplating demands good via morphology and roughness and presents challenges in filling high-aspect-ratio through-holes. Blind holes facilitate seed-layer filling, allowing electroplating filling and back thinning to produce vertical interconnection. The TGV created by electroplating generally employs copper as a conductor, with minimal interconnect resistance, rendering it a good fit for applications that require high-frequency, high-speed, and three-dimensional packaging. The magnetic assembly method boasts a low process cost that permits the attainment of low-resistance and low-stress vertical interconnection. Nevertheless, regulating the magnetic force of conductive wires poses a challenge. Vias of different types and diameters require multiple magnets to achieve internal fixation. In addition, the process reliability of repeatedly filling the SOG is not high. The comparison of TGV-metallization processes is shown in Table 2.

Glass substrates, such as BF33 and D263T borosilicate glass, are predominantly used in sensor packaging for both sealing and interconnecting purposes. These substrates possess excellent bonding performance and can be interconnected using TGV technology. Photoelectric and thermoelectric sensors can utilize the glass substrate’s high transparency and low thermal conductivity to enhance device performance, while motion and pressure sensors can leverage the substrate’s mechanical properties and sealing capability for greater device reliability. The outstanding dielectric and loss properties of glass can significantly reduce transmission loss, thereby expanding the range of sensor applications in the high-frequency and high-speed domains [108]. However, sensor packaging does not typically require high interconnection density or high-aspect-ratio interconnections at present, and there are few high-frequency and low-loss transmission applications. Therefore, mechanical drilling and chemical processing can be used to process vias, and electrical interconnection can be achieved by paste filling, evaporation plating, or conformal electroplating. The bonding and sealing performance notably affects device reliability and efficiency. Therefore, many MEMS sensors utilize glass reflow technology to fulfill the need for vertical interconnection and bonding, negating the need for extra electroplating and metallization procedures.

## 5. Conclusions

This article reviews the TGV process and sensor packaging applications. The TGV process comprises a glass through-hole manufacturing process and metallization process, and commonly used processing technologies are presented. The presented techniques for producing glass through-holes include abrasive spray processing, electrochemical discharge processing, laser ablation, photosensitive glass, glass reflow processing, and laser-induced deep etching. The introduced TGV-metallization techniques are the conductive paste filling method, electroplating method, and magnetic assembly method. We separately present the packaging applications of TGV technology by sensor type and describe the TGV process flow and capabilities in detail. This article helps with the selection of the appropriate TGV process according to the processing requirements of the sensor. The development of drilling techniques, such as laser ablation and laser-induced deep etching, as well as metallization techniques, such as electroplating and conductive paste filling, has enabled TGV technology to move toward smaller diameters, higher aspect ratios, higher densities, and more reliable interconnections. This provides higher performance solutions for heterogeneous integration, 3D stacking, and high-speed interconnection. TGV technology can be employed for the future development of sensor packaging toward smaller sizes, higher densities, and higher degrees of reliability in 3D integration, exhibiting promising potential for application.

## Figures and Tables

**Table 1:** The comparison of TGV-formation processes.

Process	AJM	EDM/ECDM	Photosensitive Glass	Glass Reflow Process	LD	LIDE
Minimum size	50 µm	20 µm	1 µm	1 µm	5 µm	5 µm
Mass fabrication efficiency	Medium	Medium	High	Low	High	High
Supported TGV array density	Low	Medium	High	Medium	High	High
TGV reliability	Medium	Low	High	High	Low	High
Complexity	Low	Medium	High	High	Low	Medium

**Table 2:** The comparison of TGV-metallization processes.

Process	Conductive Paste Filling	Magnetic Self-Assembly	Electroplating
Minimum size	20 µm	70 µm	3 µm
Aspect ratio	>10	2	15
Reliability	Low	High	High
Complexity	Low	Medium	Medium

## Data Availability

The data and code used to support the findings of this study are available from the author upon request (36120211150419@stu.xmu.edu.cn).
